# Autonomous nervous system responses to environmental‐level exposure to 5G's first deployed band (3.5 GHz) in healthy human volunteers

**DOI:** 10.1113/EP092083

**Published:** 2024-10-15

**Authors:** Layla Jamal, Lisa Michelant, Stéphane Delanaud, Laurent Hugueville, Paul Mazet, Philippe Lévêque, Tamara Baz, Véronique Bach, Brahim Selmaoui

**Affiliations:** ^1^ Department of Experimental Toxicology and Modeling (TEAM) Institut National de l'Environnement Industriel et des Risques (INERIS) Verneuil‐en‐Halatte France; ^2^ PériTox Laboratory (UMR_I 01), INERIS/UPJV INERIS Verneuil en Halatte France; ^3^ PériTox laboratory (UMR_I 01), UPJV/INERIS University of Picardy Jules Verne Amiens France; ^4^ Paris Brain Institute (ICM) Center for NeuroImaging Research (CENIR) Sorbonne University, INSERM U1127, CNRS UMR7225, Pitié‐Salpêtrière Hospital Paris France; ^5^ Department of Electromagnetic Compatibility Technical Center for Mechanical Industries (CETIM) Senlis France; ^6^ RF and Printed ELectronics for Telecom and Energy team University of Limoges, CNRS, XLIM, UMR 7252 Limoges France

**Keywords:** autonomous nervous system, event‐related responses, fifth generation, radio frequencies, skin conductance, thermal effects

## Abstract

Following the global progressive deployment of 5G networks, considerable attention has focused on assessing their potential impact on human health. This study aims to investigate autonomous nervous system changes by exploring skin temperature and electrodermal activity (EDA) among 44 healthy young individuals of both sexes during and after exposure to 3.5 GHz antenna‐emitted signals, with an electrical field intensity ranging from 1 to 2 V/m. The study employed a randomized, cross‐over design with triple‐blinding, encompassing both ‘real’ and ‘sham’ exposure sessions, separated by a maximum interval of 1 week. Each session comprised baseline, exposure and postexposure phases, resulting in the acquisition of seven runs. Each run initiated with a 150 s segment of EDA recordings stimulated by 10 repeated beeps. Subsequently, the collected data underwent continuous decomposition analysis, generating specific indicators assessed alongside standard metrics such as trough‐to‐peak measurements, global skin conductance and maximum positive peak deflection. Additionally, non‐invasive, real‐time skin temperature measurements were conducted to evaluate specific anatomical points (hand, head and neck). The study suggests that exposure to 3.5 GHz signals may potentially affect head and neck temperature, indicating a slight increase in this parameter. Furthermore, there was a minimal modulation of certain electrodermal metrics after the exposure, suggesting a potentially faster physiological response to auditory stimulation. However, while the results are significant, they remain within the normal physiological range and could be a consequence of an uncontrolled variable. Given the preliminary nature of this pilot study, further research is needed to confirm the effects of 5G exposure.

## Abstract (highlights)

- **What is the central question of this study?**
  Does autonomous nervous system activity, represented by skin temperature and electrodermal activity, change during and after exposure to 3.5 GHz antenna‐emitted signals typical of 5G mid‐band frequencies?
- **What is the main finding and its importance?**
  Head temperature significantly increased after exposure to 3.5 GHz signals, while neck temperature rose both during and after the exposure. Furthermore, a subtle but significant change in certain electrodermal parameters (CDA. Tonic activity as well as CDA and TTP Lateny) was detected following exposure, suggesting a potentially faster physiological response to auditory stimuli. However, while the findings are noteworthy, they remain within the normal physiological range and could be influenced by an uncontrolled factor.

## 1 INTRODUCTION

Fifth generation (5G) radio frequencies (RF) represent a significant leap forward, promising faster data transmission rates, enhanced network capacity, and reduced latency while using wireless communication systems. These remarkable capacities rely on mid‐ to high‐frequency bands within the RF spectrum (100 kHz to 300 GHz). 5G mid‐band frequencies typically oscillate around 3.5 GHz and have been globally and progressively introduced in urban areas for public use since 2019. On the other hand, millimetre waves around 26 GHz correspond to 5G high‐frequency bands that will be deployed later. Their shorter wavelengths support ultra‐fast data rates, making them ideal for dense urban environments and high‐capacity scenarios.

While society is eagerly embracing the benefits of this new technology, questions have been raised about its potential impact. To date, research has been extensively conducted to examine the health effects of earlier generations of RF, mostly focusing on thermal as well as non‐thermal responses. Thermal effects are simply defined by increased tissue temperature when RF energy is absorbed by the body. However, the International Commission on Non‐Ionizing Radiation Protection (ICNIRP) has established guidelines to ensure that RF exposure levels remain below the threshold at which significant harmful thermal effects such as burns or cataracts manifest (ICNIRP, 1998, 2010, 2020).

Non‐thermal effects, on the other hand, occur at RF exposure levels that do not induce significant temperature rises. These effects are less understood but have been reported in numerous studies, suggesting potential but controversial biological responses, particularly those related to the autonomic nervous system (ANS). Through its sympathetic and parasympathetic branches, the ANS maintains physiological homeostasis, including digestion, heart rate, blood pressure, and temperature regulation, among other vital functions. Body temperature regulation, a fundamental aspect of thermoregulation, is linked to ANS functioning by the hypothalamus. To maintain a stable internal temperature, the latter receives signals from peripheral thermoreceptors and then orchestrates the appropriate physiological responses. Consequently, any imbalance in the sympathetic and parasympathetic activities of the ANS can potentially disrupt the thermoregulatory processes (Tansey & Johnson, 2015).

Despite claims that RF within regulatory exposure levels does not cause temperature disruption, several animal studies (Arendash et al., 2012, 2010; Maalouf et al., 2023; Mai et al., 2021, 2020; Pelletier et al., 2013) and human studies (Bauer et al., 2018; Bortkiewicz et al., 2012; Loughran et al., 2019; Tahvanainen et al., 2007) have demonstrated the opposite.

Likewise, another non‐invasive physiological marker of ANS functioning is electrodermal activity (EDA), also known as galvanic skin response (GSR). This indicator measures the electrical conductance of the skin, primarily influenced by sympathetic nervous system activity. Thus, researchers also monitored this parameter to gain insights into the possible RF‐induced influence on the interplay between the sympathetic and parasympathetic branches of the ANS. In studies investigating skin conductance indicators in healthy human volunteers, the outcomes are controversial, showing either a significant change (Esen & Esen, 2006) or no effect at all (Selmaoui et al., 2018). In addition, other research explored the impact of RF on EDA in individuals suffering from electromagnetic hypersensitivity (EHS), where various non‐specific symptoms (e.g., fatigue, nausea, heart palpitations) and discomfort are experienced in response to exposure to electromagnetic field devices and electronics. No significant RF influences were detected in this case (Andrianome et al., 2017; Eltiti et al., 2009; Wilen et al., 2006). Nonetheless, a recent study (Bräscher et al., 2020) concluded that a nocebo effect is implicated in EHS individuals who only received sham RF exposures, perceived as genuine by the tested subjects.

Overall, these findings reinforce the hypothesis of a potential physiological regulatory mechanism upon RF exposure below the international safety thresholds. However, the results of these studies remain inconclusive and could be influenced by several factors, including heterogeneous study designs, exposure systems and, most importantly, individual perception of RF. Nonetheless, there is a notable lack of research specifically examining the effects of 5G on ANS functioning. While numerous studies, as presented above, have explored the impact of RF on ANS parameters, most of this work has focused on earlier generations of wireless technology. Given the unique characteristics of 5G networks, including higher frequency bands and increased data transmission rates, it is crucial to investigate whether these advancements may have any implications for ANS regulation. Consequently, the current study aims to assess this aspect by measuring temperature and EDA in healthy young volunteers during and after two genuine and sham exposure sessions to 3.5 GHz, representing the first deployed band of 5G networks, with simulated exposure levels (∼2 V/m) equal to those currently found in the environment according to recent dosimetry studies (Hinrikus et al., 2022; Selmaoui et al., 2021). Additionally, electrical brain and cardiac activities were also monitored. However, the outcomes of the latter parameters are presented in other publications. Here, we focus on the temperature and GSR outcomes.

## 2 METHODS

### 2.1 Ethical approval

Ethical approval for the experimental protocol (ID‐RCB no: 2020‐A03127‐32) was obtained from the French national ethical committee ‘CPP Sud‐Ouest et Outre‐Mer 1’ and adhered to the principles of the *Declaration of Helsinki*.

### 2.2 Volunteers and inclusion criteria

Our study included 44 volunteers (24 males and 20 females) who met rigorous inclusion criteria. The participants had a mean ± SD age of 26.5 ± 4.8 years and a mean ± SD body mass index of 22.6 ± 3.6 kg/m^2^. They were carefully selected to maintain regular sleeping patterns from 23.00 to 08.00 h ±1 h and had no chronic or acute illnesses or disabilities. They were non‐smokers and did not exhibit EHS. Drug or narcotic users were excluded based on the NarcoCheck urinary test (ref: DOA‐M10‐3B, Kappa City Biotech SAS, Montluçon, France). Female participants were required to have regular menstrual cycles lasting between 25 and 32 days and not be using hormonal contraception. Inclusion sessions were scheduled during the follicular phase of their menstrual cycles. Nursing or pregnant women were excluded from this study, where a pregnancy urinary test (NADAL hCG, ref: 152002, nal von minden GmbH, Moers, Germany) was performed on all female participants to confirm the corresponding criterion. However, sex type was self‐declared and reported by participants.

Additionally, volunteers were explicitly instructed to abstain from consuming any beverages or substances that can disturb the nervous system, such as caffeine, alcohol or chocolate, for 24 h prior to each experimental session.

### 2.3 Study design and experimental protocol

The current study employed a triple‐blind design. Before participation, all selected individuals provided written informed consent, indicating their voluntary agreement to participate in the study. Subsequently, participants underwent two exposure sessions, randomly assigned, and counterbalanced to mitigate potential order effects. These sessions were scheduled within a maximum interval of 1 week and maintained consistent time frames, either in the morning (09.00–11.00 h ±30 min) or in the afternoon (14.00–16.00 h ±30 min). This scheduling approach aimed to minimize potential disruptions to the participants' circadian rhythm, thereby reducing confounding variables that may influence the study outcomes.

**FIGURE 1:**
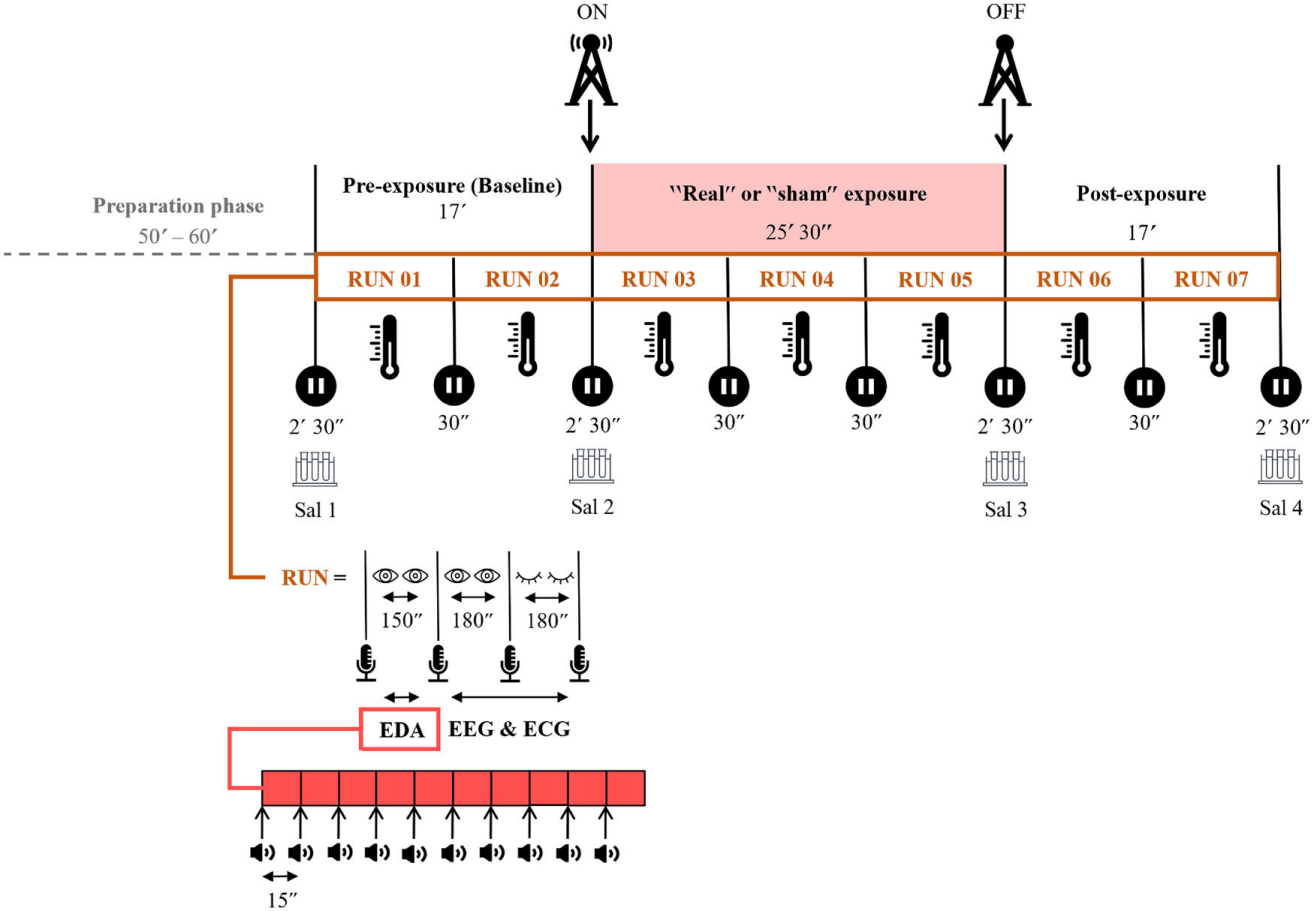
Study design and methodology. Hand, head and neck temperatures (°C) were measured throughout each session. EDA was recorded in 150 s segments during both ‘real’ and ‘sham’ sessions, with participants in an eyes‐open state. Event‐related SCRs were elicited through 10 consecutive 0.3 s auditory beeps (at 60 dB and 1000 Hz sampling rate). ECG and EEG were continuously monitored under both eyes‐open and eyes‐closed conditions. Prerecorded vocal instructions guided volunteers to open and close their eyes. This article exclusively discusses the findings related to EDA and temperature. ECG, electrocardiographs; EDA, electrodermal activity; EEG, electroencephalographs; SCR, skin conductance responses.

In each session, seven runs were acquired. Each run lasted for 510 s, beginning with 150 s of EDA recording while eyes were open (EO). Another segment of eyes‐open accompanied by an eyes‐closed (EC) state of 3 min for each followed to acquire the electroencephalogram (EEG) and electrocardiogram (ECG) of volunteers. These runs were partitioned into three recording periods, starting with two runs of baseline or pre‐exposure period, to establish normal parameter values. Subsequently, three runs of a ‘real’ or ‘sham’ 5G exposure period followed to end the session with two runs of the postexposure period with no radio frequency emissions to explore any potential residual effects of the 5G frequencies. Body temperature was continuously measured during the session (see Section 2.4). During each run, volunteers were in a restful seated position, and they were vocally instructed to close and open their eyes. On the other hand, the EDA recordings (see Section 2.5) included 10 consecutive auditory beeps with 15 s intervals to elicit event‐related skin conductance responses (SCRs). Each auditory stimulation lasted 0.3 s and had signal tones of 60 dB and a sampling rate of 1000 Hz (Figure 1).

In this article, we present only the skin temperature and EDA results, with the latter recorded exclusively during the eyes‐open condition. It is important to note that the experimental protocol includes distinct segments for eyes‐open and eyes‐closed conditions, as the state of the eyes has been shown to influence various physiological responses, including EEG readings (Barry & Blasio, 2017; Barry et al., 2007; Danker‐Hopfe et al., 2019; Wallace et al., 2022). To mitigate potential bias in our analysis of brain activity, the protocol was specifically designed to control for this variable. However, though skin temperature was continuously monitored during all the segments unlike the EDA, we did not control the eyes condition for this metric.

### 2.4 Exposure system

The participants were exposed to a frequency of 3.5 GHz in a controlled ambient temperature (mean ± SD: 25.6 ± 0.9°C) and humidity (mean ± SD: 41.6 ± 4.4%). The exposure occurred in a dimly lit room that was electrically shielded. The emissions were transmitted to the participants through a horn antenna (BBHA 9120 D, Schwarzbeck Mess‐Elektronik oHG, Schönau, Germany) positioned 120 cm away at a 45‐degree angle to the right of the participants. The antenna was connected to a 5G generator (SMB100A [1406.6000.02], Rohde & Schwarz GmbH & Co. KG, Munich, Germany) and a signal amplifier (SX 40/15, Prâna, Brive‐la‐Gaillarde, France), both located in a separate room. The emitted frequencies were pulse‐modulated at a modulation rate of 577 µs/4.6 ms. The electrical field intensity measured 2 V/m at the head level and 1.5 V/m at the trunk level, using a field meter (NBM 550, Narda Safety Test Solutions GmbH, Pfullingen, Germany) to mimic the current 5G exposure levels found in the environment (Hinrikus et al., 2022; Selmaouiet al., 2021). The peak power density (PD) was estimated to be 0.68 W/m^2^, while the specific absorption rate (SAR) measured 0.037 ± 0.11 mW/kg and 0.008 ± 0.019 mW/kg when averaged over the head and brain, respectively. The ‘real’ and ‘sham’ exposures were controlled using a dosimeter (MVG/EME Spy Evolution, MVG Industries | Satimo, Villejust, France) to confirm the successful blinding procedure at the end of the overall experiment. Detailed information on the exposure system can be found in our previous article discussing the 5G effects on the brain electrical activity of the studied volunteers (Jamal et al., 2023).

### 2.5 Temperature measurement and analysis

To conduct efficient and non‐invasive real‐time screening of skin temperature, we used a device‐connected probe (ESCORT Junior Body Temperature Data Logger, Cryopak Digital, Edison, NJ, USA) positioned on the dorsal side of the left hand to measure the peripheral temperature of the volunteers. The probe was shielded by a hydrogel adhesive cover (Hydrogel temperature probe cover, Ref: 3159, Médiprema, Tauxigny‐Saint‐Bauld, France) to mitigate radio frequency interference. Additionally, an infrared camera (FLIR B400 25°, France Infra Rouge, Pontachateau, France), situated 200 cm away from the participants, was employed to measure the overall body skin temperature. Subsequently, specific temperature points on the head and neck were identified using ThermaCAM Researcher software (version Pro 2.9) for further analysis. Temperature was continuously monitored throughout each session, with 30 s intervals between measurements. Ambient humidity (mean ± SD: 41.6 ± 4.4%) and temperature (mean ± SD: 25.6 ± 0.9°C) were also recorded (Alecto Mini weather station—WS100, Commaxx BV, Kerkrade, Netherlands) and used to correct the camera images in conjunction with the hand temperature recorded by the ESCORT device.

### 2.6 EDA

We placed two electrodes (TSD203 finger transducer, BIOPAC Systems, Inc., Goleta, CA, USA) on the tips of the second and third fingers of the non‐dominant hand of volunteers to measure skin conductance. The signal was amplified (BRAINAMP DC, Brain Products GmbH, Herrsching, Germany) and recorded with BrainVision Recorder software (version 1.23.0003). We then processed the acquired data with the Ledalab program (version V3.4.9) (Benedek & Kaernbach, 2010), where the sample frequency was 1000 Hz, and the response window ranged from 1 to 4 s. In addition, the amplitude threshold of SCRs was determined at 0.02 µS. We then downsampled the data to 20 Hz (50‐fold) to accelerate the analysis. Subsequently, after visual artifact inspection and data optimization, we analysed the data with the method of continuous decomposition analysis (CDA) (Benedek & Kaernbach, 2010). The latter captures the underlying Sudomotor nerve activity (SNA) of signals with improved temporal precision and deconvolves the skin conductance data by the general response shape to generate continuous phasic and tonic decomposed components (CDA.Tonic: average of tonic or skin conductance level within the response window (wrw) in microsiemens (µS); CDA.SCR: mean of phasic activity wrw (µS); CDA.Latency: the response latency of the first significant SCR wrw in seconds; CDA.nSCR: the number of significant SCRs wrw; and CDA.AmpSum: the sum of amplitudes of significant SCRs wrw in µS).

In addition, the standard trough‐to‐peak (TTP) metrics (TTP.Latency (s); TTP.nSCR and TTP.AmpSum (µS)) alongside the global skin conductance and the maximum positive deflection of response peaks were computed as well. It is noteworthy that the EDA was continuously acquired throughout the sessions in each recorded run (a total of seven runs in both ‘real’ and ‘sham’ exposure sessions). However, we were only interested in the event‐related SCRs corresponding to the auditory stimulations (a total of 10 stimuli). Meanwhile, only the first two bursts of these stimulations were considered and averaged for each run and then statistically compared as described in Section 2.6. This process was carried out to avoid any habituation effect related to repeated stimuli, which could bias the results.

### 2.7 Statistical analysis

A baseline correction was made for both temperature and EDA data, where runs 1 and 2 were averaged for each subject and session for each parameter. Then, this mean was subtracted from other runs. Concerning the EDA data, two subjects were excluded because no data were available for one of the two sessions.

Subsequently, a two‐way mixed‐effects model was employed on the baseline‐corrected data to assess the impact of two factors: time periods (five levels: runs 3, 4, 5, 6 and 7) and 5G (two levels: real and sham), as well as their interactions. Subsequently, Tukey's multiple comparison test was conducted to examine the mean differences among the groups under analysis, particularly between ‘real’ and ‘sham’ sessions. All statistical analyses were performed using R Studio software (version 4.3.3), with a predetermined significance level of 0.05 (*P* < 0.05). Furthermore, we applied a Greenhouse–Geisser correction to account for any potential non‐sphericity in the data.

Moreover, it is notable that we analysed both male and female participants collectively to uphold a requisite statistical power (>80%). For forthcoming studies, it is advisable to incorporate more volunteers to facilitate sex‐based analyses with adequate statistical robustness.

## 3 RESULTS

### 3.1 Temperature

The two‐way mixed effects model analysis of the head and neck temperature revealed significant effect related to 5G exposure (*F* (1, 7401) = 38.553; *P* < 0.0001 and *F* (1, 7483) = 155.613; *P* < 0.0001, respectively) (Supporting information, Table S1) and Tukey's multiple comparison test showed significant difference (*P* < 0.0001; Supporting information, Table S2). As for the collected temperature points on the hand, there was no significant difference observed due to 3.5 GHz exposure (*F* (1, 7291) = 0.2950; *P* = 0.5866).

Moreover, for the hand and neck temperature, a significant effect was found due to the time periods factor (runs) (*P* < 0.0001; Table S1), which was validated by Tukey's test for some runs (see Table S2).

**FIGURE 2:**
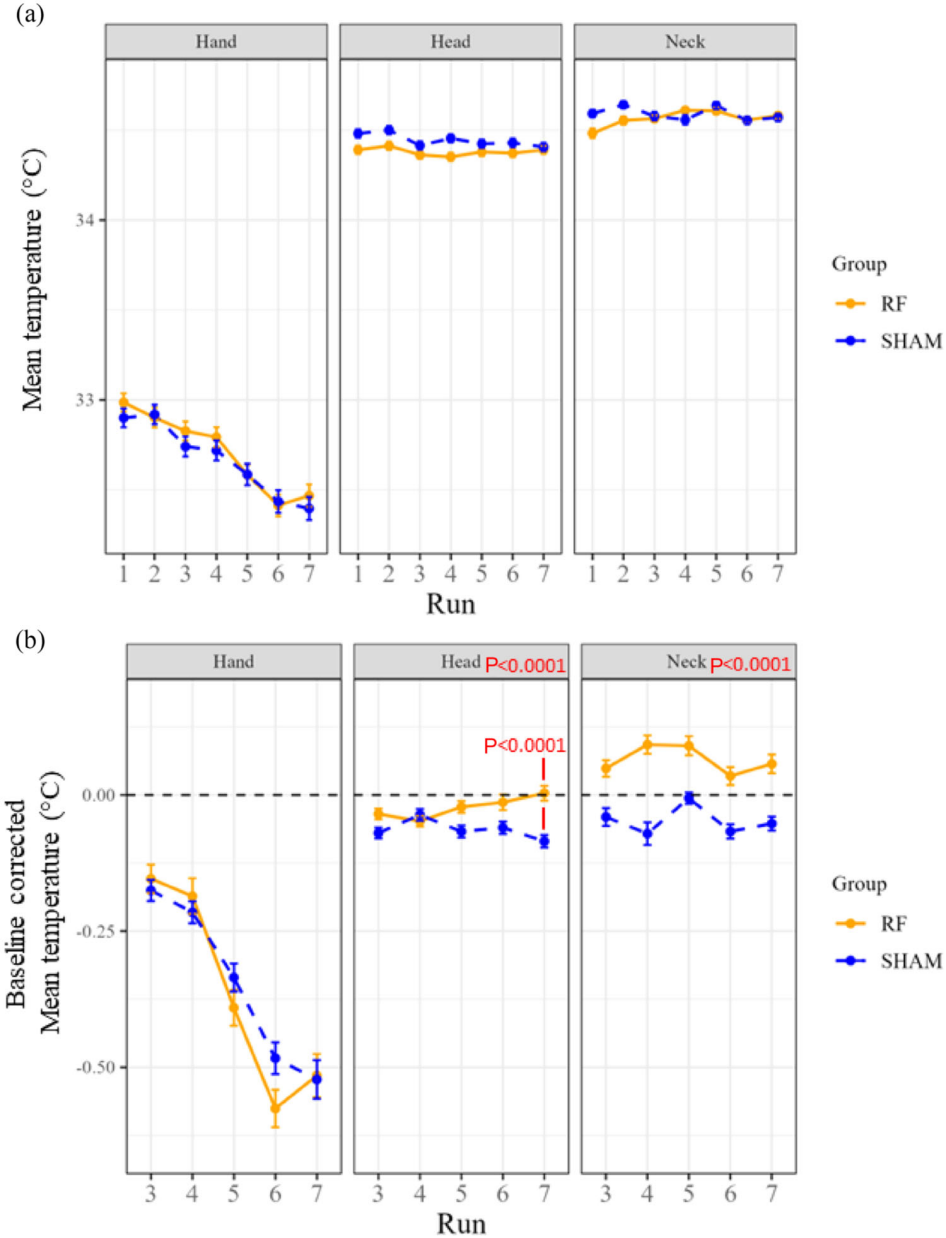
Outcomes of hand, neck, and head temperature related to each time or recording period for ‘raw values’ (a) and baseline corrected data (b). The values of participants (*n* = 44) are presented as means ± SEM (error bars). The *P*‐value corresponds to the significant effect detected by Tukey's multiple comparison test (Supporting information, Table S2).

Furthermore, the interaction between the time periods (runs) and 5G exposure factors was only significant in the hand (*P* = 0.0065) and head (*P* < 0.0001) temperature data (Table S1). Tukey's multiple comparison test showed that this significant difference is only detected in Run 07 (post‐exposure period; *P* < 0.0001) concerning the head temperature (Table S2 and Figure 2b).

The statistical outcomes of the two‐way mixed effects model are presented in Table S1, and Tukey's multiple comparison test results are shown in Table S2.

### 3.2 Analysis of skin conductance and event‐related responses of the EDA

#### 3.2.1 Findings of decomposed analysis

**FIGURE 3:**
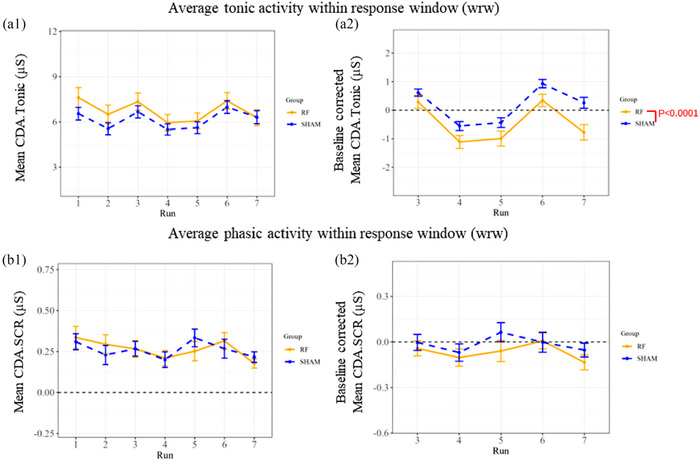
Decomposed tonic (a) and phasic (b) EDA measures during ‘real’ (RF) and ‘sham’ 5G exposure sessions. The values presented for each run in each session represent the means derived from the initial two auditory stimuli with ‘raw values’ (1) and baseline‐corrected data (2), with the SEM depicted as error bars for the included volunteers (*n* = 42). The *P*‐value corresponds to the significant effect detected by Tukey's multiple comparison test (Supporting information, Table S4).

The decomposed EDA signal revealed a significant difference in tonic activity related to 5G exposure (*P* < 0.0001) when ‘real’ and ‘sham’ sessions were compared, as indicated by the two‐way mixed effects analysis (Figure 3a2 and Supporting information, Table S3). Subsequent Tukey's multiple comparison tests demonstrated that this significance was only observed during Run 07 (post‐exposure period). In this segment, the tonic activity exhibited a notable increase in the ‘real’ exposure session compared to the sham session (*P* = 0.0136) (Supporting information, Table S4).

**FIGURE 4:**
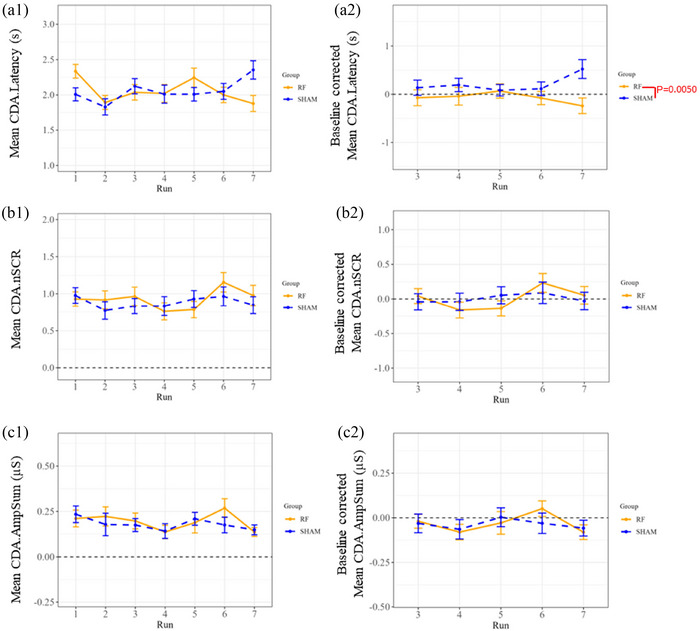
Decomposed EDA measures are computed from stimulated run segments by 10 repeated auditory beeps during ‘real’ (RF) and ‘sham’ 5G exposure sessions with ‘raw values’ (1) and baseline corrected data (2). However, only the first two stimulations of the baseline corrected data were considered for the statistics. Latency of SCRs (a), the number of significant SCRs (b), and the sum of amplitudes of significant SCRs (c) are displayed as means ± SEM (error bars) for all the volunteers (*n* = 42). Significance levels (*P*‐value) indicate 5G‐related effects identified by Tukey's multiple comparison test (Table S4).

In addition, the phasic component CDA.Latency also exhibited a significant effect for the 5G exposure factor (*P* = 0.0092) according to the 2‐way mixed effects model (see Figure 4a2 and Table S3). However, this effect was solely discernible during Run 07 (post‐exposure period) following Tukey's correction test, indicating a significant decrease (*P* = 0.0245) attributable to the ‘real’ 5G exposure compared to ‘sham’ exposure (Table S4).

On the other hand, the other phasic components of the decomposed EDA metrics (CDA.SCR, CDA.AmpSum and CDA.nSCR) did not show any significant change between the ‘real’ and ‘sham’ exposure sessions regarding 5G exposure factor (*P* = 0.1231, 0.8742 and 0.9999, respectively; see Figures 3, 4 and 4c2, respectively).

Moreover, the results of the tonic parameter (CDA.Tonic) were also significant for the time period factor between runs (*P* < 0.0001), as detailed in Table S3. Furthermore, Tukey's multiple comparison test analysis (see Table S4) confirmed these findings and also revealed a significant influence between most runs (*P* < 0.0001).

In terms of the interaction between the time periods (runs) and 5G exposure factors, no significant effect was detected in the two‐way mixed effects model for CDA.Latency (*P* = 0.1723), CDA.SCR (*P* = 0.7813), CDA.AmpSum (*P* = 0.7465), CDA.nSCR (*P* = 0.5616) and CDA.Tonic (*P* = 0.2413) (Tables S3 and S4).

#### 3.2.2 Standard TTP and global measures outcomes

**FIGURE 5:**
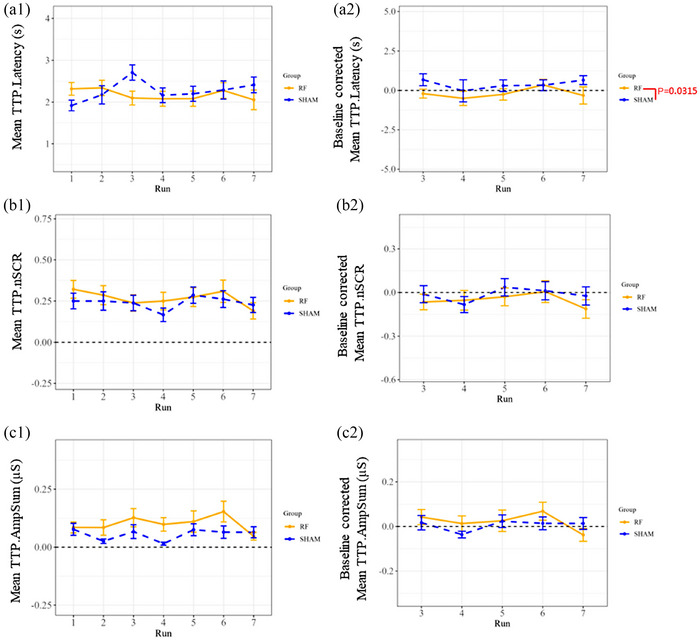
Standard TTP EDA measures are computed from stimulated run segments by 10 repeated auditory beeps during ‘real’ and ‘sham’ 5G exposure sessions with ‘raw values’ (1) and baseline corrected data (2). However, only the first two stimulations of the baseline‐corrected data were considered for the statistics. Latency of SCRs (a), the number of significant SCRs (b), and the sum of amplitudes of significant SCRs (c) are displayed as means ± SEM (error bars) for all the participants (*n* = 42). Significance levels (*P*‐value) indicate 5G‐related effects identified by Tukey's multiple comparison test (Table S4).

Exposure to 3.5 GHz did not reveal any significant variation in the TTP parameters (TTP.nSCR, *P* = 0.3274 and TTP.AmpSum, *P* = 0.45339), except for TTP.Latency (*P* = 0.0002), as shown in the two‐way mixed effects (see Figure 5a2 and Table S3). However, the subsequent Tukey's test failed to confirm this significance in all tested group combinations except for the whole group comparison (mean of all exposure and postexposure runs between the ‘real’ and ‘sham’ exposure sessions (Figure 5a2 and Table S4). However, we consider this finding to be a random outcome since the test on correspondent runs between the ‘real’ and ‘sham’ exposure sessions did not reveal any significance (Table S4).

**FIGURE 6:**
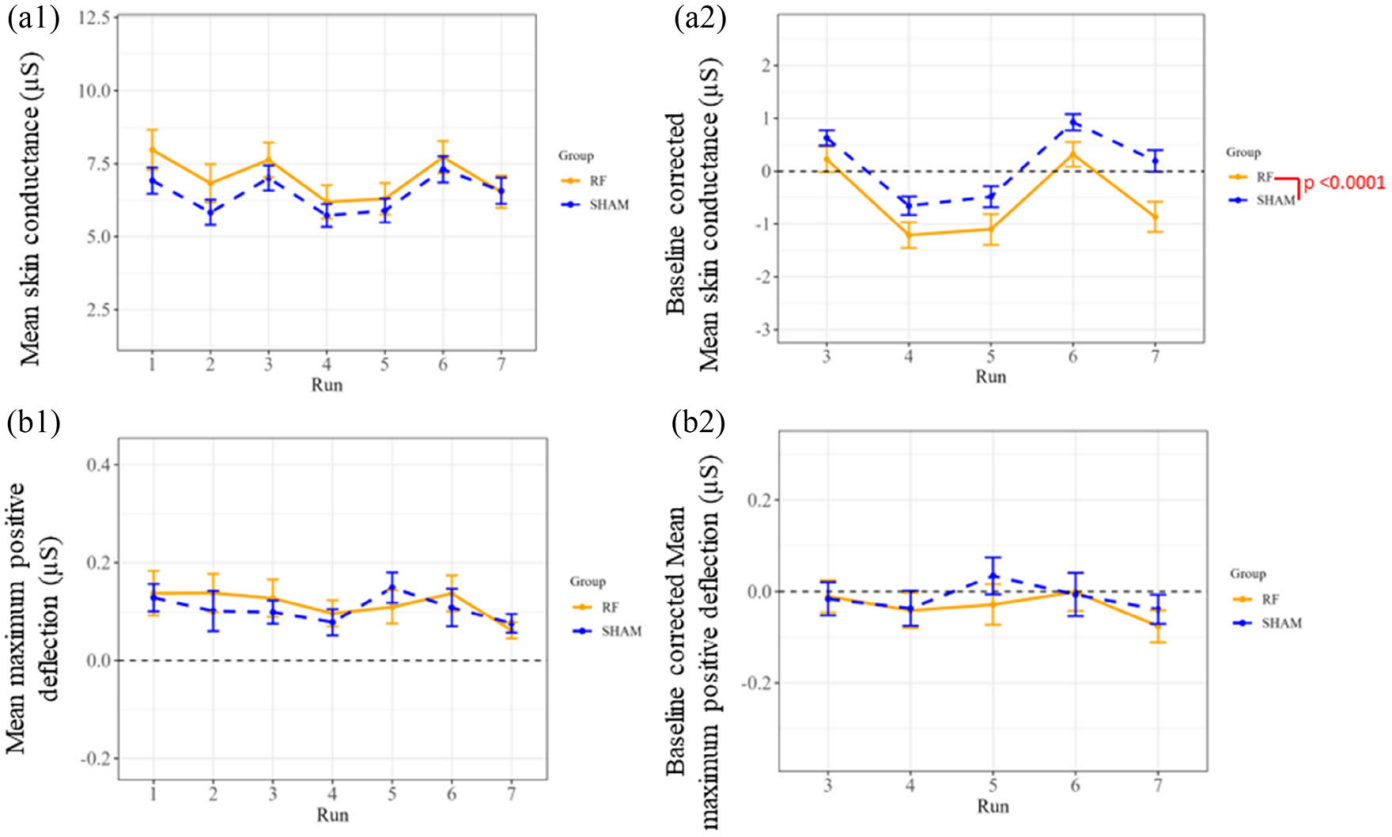
Global standard measures of skin conduction (a) and maximum positive deflection (B) computed from evoked‐EDA run segments during genuine (RF) and simulated (Sham) 5G exposure sessions with ‘raw values’ (1) and baseline corrected data (2). Data in each run represent the average values of the first two repeated auditory stimuli, with their corresponding SEM as error bars for all the participants (*n* = 42). Significance levels (*P*‐value) indicate 5G‐related effects identified by Tukey's multiple comparison test (Table S4).

Concerning the global measures of EDA metrics, only the global mean of skin conductance (SC) (*P* < 0.0001) showed a significant effect due to 5G exposure in the two‐way mixed effects analysis (see Figure 6 and Table S3), where Run 07 (post‐exposure period) was the only segment that exhibited a significant effect (*P* = 0.0285) following Tukey's multiple comparison test (Table S4). This parameter also displayed significant alterations attributed to the time period (runs) in both mixed effects model factor (*P* < 0.0001) (Table S3) and Tukey's multiple comparison test (Table S4). However, no other parameter showed a significant effect for the time period (runs) factor or its interaction with the 5G exposure (see Table S3).

## 4 DISCUSSION

The current study aimed to assess the first globally deployed 5G band at 3.5 GHz for any potential impact on the ANS. For this purpose, skin temperature and induced EDA were evaluated in 44 healthy human volunteers at a controlled ambient temperature using pulse‐modulated antenna emissions as an exposure system. The exposure level (2 V/m and 1.5 V/m at the head and trunk levels, respectively) was simulated to mimic the regulated current 5G scenario found in the environment, as presented in recent publications (Hinrikus et al., 2022; Selmaoui et al., 2021). Each participant took part in two randomized, counterbalanced and blinded sessions, each containing baseline and postexposure periods with no RF. These periods were separated by either a genuine or a sham exposure phase, lasting 25 min and 30 s.

Skin temperature was measured by a device‐connected probe placed on the dorsal side of the left hand, alongside an infrared camera that measured trunk temperature. Later, only two anatomical points of the head and neck were analysed in addition to hand temperature. Exposure to 5G did not change the skin temperature of the hand. However, the head and neck temperatures showed a minimal significant temperature difference between the genuine 5G and sham exposure sessions (an increase in the average temperature at Run 07 of the postexposure period for the head when compared to the ‘sham’ exposure session along with the average neck temperature, which also showed an increase in all the exposure (Run 03, Run 04, and Run 05) and post‐exposure (Run 06 and Run 07) ‘real’ 5G periods versus those of ‘sham’). However, the temperature increase in the head and neck could be explained by the fact that the main beam of the antenna was directed toward these body parts and consequently received the maximum intensity (2 V/m) while the hands were placed on the table and received less than 1 V/m. It should be noted that 2 V/m is not a level of electromagnetic field which could cause thermal effects, but it can challenge the biological system without consequences since the body can react to regulate the temperature (thermoregulation). This phenomenon has been observed in animal studies with a 2G or 3G signal. Indeed a series of animal research on rodents found an increase in body temperature after chronic RF exposure (Arendash et al., 2012, 2010; Mai et al., 2020), suggesting a thermogenesis effect. Subsequently, time‐ and dose‐dependent RF responses were found in brown and white adipose tissues of exposed mice, where the mRNA expression of certain thermogenesis‐implicated genes was altered after 3 h of exposure then partially compensated after 7 h (Maalouf et al., 2023).

Regarding human studies of RF‐induced temperature effects, there are no experimental studies for long‐term exposures, as far as we know. However, those of short‐term (≤30 min) using regulatory exposure limits demonstrate controversial thermal‐related RF effects. In some studies, tympanic temperature in healthy young adults was elevated due to 900 or 1800 MHz exposures (Bauer et al., 2018; Bortkiewicz et al., 2012; Tahvanainen et al., 2007), while in other studies, non‐significant increased trends were detected (Lindholm et al., 2011; Schneider, 2022). Other research investigating mobile‐phone RF (900 MHz) impacts on skin temperature did not observe any significant changes (Ghosn et al., 2012; Loos et al., 2013). On the other hand, a recent study (Loughran et al., 2019) showed that antenna‐emitted RF (920 MHz) significantly increased skin temperature in a dose‐dependent manner, with a specific absorption rate (SAR) of 1 W/kg, while an exposure level of 2 W/kg had an impact of an elevated non‐significant trend. The authors suggested a potential thermoregulatory response according to RF exposure levels. Nevertheless, whether or not low RF (<6 GHz) produces thermal effects depends on several factors such as frequency, intensity, signal type (pulsed or continuous), duration of exposure and type of exposed organs, explaining the difficulty of comparing protocols and existing literature results with those of higher RF millimetric waves (>6 GHz). The latter have a less or non‐penetrative nature in living organisms, making their interaction less complex. Thus, their thermal effects have been well‐established and documented compared to lower RF (<6 GHz), as explained by several reviews and reports (Adair & Black, 2003; ICNIRP, 1998, 2020; SCENIHR, 2015). Consequently, our current findings and the above‐mentioned studies highlight the importance of further research to understand the biological mechanisms underlying these result differences.

In terms of EDA, we were interested in exploring the event‐related skin responses (SCRs) induced by 10 repeated auditory bursts for 150 s in seven recorded runs (only the first two stimulations were considered for the statistical analysis). The global mean of skin conductance along with the tonic activity and latency of the decomposed EDA metrics were significant in response to 5G. The change in the global mean of skin conductance suggests that exposure to 5G may influence the physiological response to auditory stimulus even within normal ranges. Furthermore, a decrease in latency may suggest a faster or more efficient cognitive response with an increase in processing speed and a reduction in the time needed to make decisions with perhaps fewer mental resources. However, the conventional TTP latency parameter was not significant after statistical correction of the outcomes. In addition, there was a significant difference in the tonic activity and global means of skin conductance due to time. Consequently, this preliminary study shows that 5G exposure within the environmental levels seems to affect some parameters of EDA that are more pronounced after the exposure. However, future studies should be conducted to validate these outcomes.

Similar to the skin temperature findings, we currently cannot compare these results with other research studies. However, a previous study performed on healthy young participants who were exposed to mobile‐phone GSM signals (900 MHz) did not display any elicited RF‐related differences in the stimulated EDA parameters (Selmaoui et al., 2018). Nevertheless, the same significant changes were found regarding tonic activity and global skin conductance in the ‘real’ exposure sessions. Yet, it was concluded that these outcomes are not attributed to 2G exposure since the baseline pre‐exposure periods also exhibited the same elevated levels of these parameters. On the other hand, only one study (Esen & Esen, 2006) reported a decreased latency response in exposed healthy volunteers to mobile GSM frequencies (900 MHz), suggesting slower reactions for mobile phone users, and thus potential risks to external stimulations. Nevertheless, neither atopic dermatitis (Johansson et al., 2008) nor electromagnetic‐hypersensitive individuals (EHS) exposed to antenna (Andrianome et al., 2017; Wilen et al., 2006) or mobile base station (Eltiti et al., 2009) GSM signals revealed any significant effects on the skin conductance parameters. Interestingly, a recent study (Bräscher et al., 2020) on EHS volunteers observed a significant effect on SCRs due to sham Wi‐Fi (2.45 GHz) exposure, indicating a potential nocebo psychological effect in these individuals.

### 4.1 Conclusion

Our study suggests that exposure to 3.5 GHz signals may affect head temperature after exposure and neck temperature both during and after exposure. To confirm these effects, future studies should replicate our findings. Additionally, our analysis of EDA shows a statistically significant decrease in overall mean skin conductance and changes in the decomposed tonic and latency EDA components following exposure to 5G. This suggests a potential modulation of physiological responses to auditory stimuli by 5G exposure, possibly indicating faster cognitive processing and decision‐making. However, since these observed differences remain within normal physiological ranges, further investigation is needed to validate the findings and account for any uncontrolled variables.

It is important to note that our findings are specific to our experimental conditions (short term exposure, and the low intensity around 2 V/m), corresponding to the current environmental exposure to 5G. Nonetheless, our results underscore the urgency of continued inquiry into the biological mechanisms underlying these physiological changes and in the long‐term exposure. Such research is crucial for informing health authorities in the formulation and revision of policies and guidelines governing the responsible usage of emerging technologies.

## AUTHOR CONTRIBUTIONS

Layla Jamal: Methodology; investigation; data curation; formal analysis; visualization; writing—original draft. Lisa Michelant: Methodology; investigation; data curation; formal analysis; visualization; writing—review and editing. Stéphane Delanaud: Methodology; resources; validation (temperature data). Laurent Hugueville: Methodology; resources; software; validation (data of electrodermal activity). Paul Mazet: Methodology (exposure system). Philippe Leveque: Methodology; investigation; formal analysis (exposure system). Tamara Baz: Formal analysis; visualization. Véronique Bach: Supervision; data curation; formal analysis (temperature data). Brahim Selmaoui: Conceptualization; funding acquisition; methodology; project administration; validation; resources; writing—review and editing, supervision. All authors have read and approved the final version of this manuscript and agree to be accountable for all aspects of the work in ensuring that questions related to the accuracy or integrity of any part of the work are appropriately investigated and resolved. All persons designated as authors qualify for authorship, and all those who qualify for authorship are listed.

## CONFLICT OF INTEREST

The authors and their collaborators declare no conflicts of interest.

## Supporting information



Tables S1–S4.

## Data Availability

The entire raw dataset will be hosted on a not‐for‐profit repository of the European consortium for the GOLIAT project: https://projectgoliat.eu/publications/
