# Correction to “Autonomous nervous system responses to environmental‐level exposure to 5G's first deployed band (3.5 GHz) in healthy human volunteers”

**DOI:** 10.1113/EP093357

**Published:** 2025-10-13

Jamal, L., Michelant, L., Delanaud, S., Hugueville, L., Mazet, P., Lévêque, P., Baz, T., Bach, V., & Selmaoui, B. (2024). Autonomous nervous system responses to environmental‐level exposure to 5G's first deployed band (3.5 GHz) in healthy human volunteers. *Experimental Physiology*, *109*(12), 2122–2133. https://doi.org/10.1113/EP092083

The original article should have included a section titled ‘Funding information’, with the following text:

‘This project has received funding from the European Union's Horizon Europe research and innovation programme under grant agreement No 101057262. Views and opinions expressed are however those of the authors only and do not necessarily reflect those of the European Union or the Health and Digital Executive Agency. Neither the European Union nor the granting authority can be held responsible for them.’

We apologize for this error.

