# Supplementary material for: Autonomous nervous system responses to environmental‐level exposure to 5G's first deployed band (3.5 GHz) in healthy human volunteers
Source: Exp Physiol. 2024 Oct 15;109(12):2122–33. doi: 10.1113/EP092083 (PMC11607616; doi:10.1113/EP092083)
Supplement: Supplementary file 1 — Tables S1–S4. [file EPH-109-2122-s001.docx]

# **Supplementary materials**

**Table S1.** Statistical results of the participants (n = 44) from 2-way mixed effects model examining time periods and 5G factors on hand, neck, and head temperature are presented. “Dfn” and “DFd” represent the degrees of freedom for the numerator and denominator of the F ratio, respectively. Significant findings (p < 0.05) are highlighted in green.

| **Fixed effects (F-value (DFn, DFd); p-value):** | **Time periods** | **5G exposure** | **Interaction** |  |
| --- | --- | --- | --- | --- |
| **Hand** | F (4, 7288) = 131.21; p < 0.0001 | F (1, 7291) = 0.29; p = 0.5866 | F (4, 7283) = 3.56; p = 0.0065 |  |
| **Neck** | F (4, 7480) = 4.75; p < 0.0001 | F (1, 7483) = 155.61; p < 0.0001 | F (4, 7475) = 2.36; p = 0.0509 |  |
| **Head** | F (4, 7398) = 0.77; p = 0.5411 | F (1, 7401) = 38.55; p < 0.0001 | F (4, 7393) = 7.23; p < 0.0001 |  |

**Table S2.** The results of Tukey’s comparison test between the real “RF” and Sham exposure sessions for hand, neck and head temperature, calculated from the IR camera, are presented for all the included volunteers (n = 44). Significant p-values (< 0.05) are highlighted in green (group comparisons between runs (time periods) within each session) and red (correspondent run comparison between the real “RF” and sham sessions). Abbreviations: CI, confidence interval.

| **Component** | **Tukey's multiple comparisons test** | **Mean Difference** | **95.00% CI of difference** | **Adjusted p-value** |
| --- | --- | --- | --- | --- |
| Hand | SHAM-RF | 0.01796235 | -0.01943084 0.05535555 | 0.3464 |
|  | Run4-Run3  Run5-Run3  Run6-Run3  Run7-Run3  Run5-Run4  Run6-Run4  Run7-Run4  Run6-Run5  Run7-Run5  Run7-Run6 | -0.03594249  -0.19797977  -0.36450983  -0.35400383  -0.16203729  -0.32856734  -0.31806135  -0.16653006  -0.15602406  0.01050600 | -0.11825356 0.04636859  -0.28022037 -0.11573918  -0.44669435 -0.28232531  -0.43623038 -0.27177728  -0.24443222 -0.07964235  -0.41090630 -0.24622838  -0.40044226 -0.23568043  -0.24879856 -0.08426155  -0.23833456 -0.07371356  -0.07174847 0.09276046 | 0.7563  < 0.0001  < 0.0001  < 0.0001  < 0.0001  < 0.0001  < 0.0001  < 0.0001  < 0.0001  0.9968 |
|  | Run4:RF-Run3:RF  Run5:RF-Run3:RF  Run6:RF-Run3:RF  Run7:RF-Run3:RF  Run3:SHAM-Run3:RF  Run4:SHAM-Run3:RF  Run5:SHAM-Run3:RF  Run6:SHAM-Run3:RF  Run7:SHAM-Run3:RF  Run5:RF-Run4:RF  Run6:RF-Run4:RF  Run7:RF-Run4:RF  Run3:SHAM-Run4:RF  Run4:SHAM-Run4:RF  Run5:SHAM-Run4:RF  Run6:SHAM-Run4:RF  Run7:SHAM-Run4:RF  Run6:RF-Run5:RF  Run7:RF-Run5:RF  Run3:SHAM-Run5:RF  Run4:SHAM-Run5:RF  Run5:SHAM-Run5:RF  Run6:SHAM-Run5:RF  Run7:SHAM-Run5:RF  Run7:RF-Run6:RF  Run3:SHAM-Run6:RF  Run4:SHAM-Run6:RF  Run5:SHAM-Run6:RF  Run6:SHAM-Run6:RF  Run7:SHAM-Run6:RF  Run3:SHAM-Run7:RF  Run4:SHAM-Run7:RF  Run5:SHAM-Run7:RF  Run6:SHAM-Run7:RF  Run7:SHAM-Run7:RF  Run4:SHAM-Run3:SHAM  Run5:SHAM-Run3:SHAM  Run6:SHAM-Run3:SHAM  Run7:SHAM-Run3:SHAM  Run5:SHAM-Run4:SHAM  Run6:SHAM-Run4:SHAM  Run7:SHAM-Run4:SHAM  Run6:SHAM-Run5:SHAM  Run7:SHAM-Run5:SHAM  Run7:SHAM-Run6:SHAM | -0.03176690  -0.23666691  -0.42151088  -0.36107432  -0.02123434  -0.06143574  -0.18120092  -0.32926389  -0.36825807  -0.20490001  -0.38974398  -0.32930742  0.01053256  -0.02966884  -0.14943402  -0.29749699  -0.33649117  -0.18484397  -0.12440741  0.21543257  0.17523117  0.05546599  -0.09259698  -0.13159116  0.06043656  0.40027654  0.36007514  0.24030996  0.09224699  0.05325281  0.33983998  0.29963858  0.17987340  0.03181043  -0.00718375  -0.04020141  -0.15996658  -0.30802955  -0.34702373  -0.11976518  -0.26782815  -0.30682233  -0.14806297  -0.18705715  -0.03899418 | -0.16708202 0.103548217  -0.37212194 -0.101211885  -0.55668710 -0.286334665  -0.49643596 -0.225712680  -0.15599976 0.113531079  -0.19661196 0.073740471  -0.31601155 -0.046390290  -0.46416527 -0.194362515  -0.50311402 -0.233402124  -0.34044759 -0.069352430  -0.52501294 -0.254475019  -0.46476168 -0.193853160  -0.12432588 0.145391008  -0.16493780 0.105600118  -0.28433764 -0.014530392  -0.43249130 -0.162502680  -0.47144008 -0.201542257  -0.32025288 -0.049435054  -0.26000144 0.011186614  0.08043375 0.350431399  0.03982225 0.310640083  -0.07957797 0.190509952  -0.22773153 0.042537570  -0.26668036 0.003498039  -0.07487894 0.195752058  0.26555747 0.534995613  0.22494513 0.495205145  0.10554566 0.375074259  -0.04260808 0.227102065  -0.08155682 0.188062440  0.20493486 0.474745111  0.16432308 0.434954078  0.04492311 0.314823695  -0.10323051 0.166851375  -0.14217931 0.127811813  -0.17492048 0.094517664  -0.29431882 -0.025614341  -0.44247285 -0.173586257  -0.48142145 -0.212626020  -0.25452947 0.014999123  -0.40268322 -0.132973072  -0.44163196 -0.172012696  -0.28255159 -0.013574356  -0.32150020 -0.052614104  -0.17352823 0.095539861 | 0.9992  < 0.0001  < 0.0001  < 0.0001  0.9999  0.9152  0.0008  < 0.0001  < 0.0001  < 0.0001  < 0.0001  < 0.0001  0.9999  0.9995  0.0165  < 0.0001  < 0.0001  0.0006  0.1046  < 0.0001  0.0017  0.9539  0.4788  0.0636  0.9234  < 0.0001  < 0.0001  < 0.0001  0.4814  0.9640  < 0.0001  < 0.0001  0.0010  0.9992  1.0000  0.9949  0.0064  < 0.0001  < 0.0001  0.1322  < 0.0001  < 0.0001  0.0178  0.0004  0.9959 |
| Neck | SHAM-RF | -0.1119989 | -0.1317267 -0.09227114 | < 0.0001 |
|  | Run4-Run3  Run5-Run3  Run6-Run3  Run7-Run3  Run5-Run4  Run6-Run4  Run7-Run4  Run6-Run5  Run7-Run5  Run7-Run6 | 0.006647834  0.038474583  -0.020790499  -0.002600114  0.031826748  -0.027438333  -0.009247948  -0.059265081  -0.041074696  0.018190385 | -0.036633274 0.049928943  -0.004893748 0.081842914  -0.064093299 0.022512301  -0.045939235 0.040739008  -0.011655477 0.075308974  -0.070855199 0.015978533  -0.052701040 0.034205144  -0.102768898 -0.015761265  -0.084614666 0.002465274  -0.025284313 0.061665083 | 0.9935  0.1098  0.6850  0.9998  0.2674  0.4189  0.9779  0.0018  0.0753  0.7843 |
|  | Run4:RF-Run3:RF  Run5:RF-Run3:RF  Run6:RF-Run3:RF  Run7:RF-Run3:RF  Run3:SHAM-Run3:RF  Run4:SHAM-Run3:RF  Run5:SHAM-Run3:RF  Run6:SHAM-Run3:RF  Run7:SHAM-Run3:RF  Run5:RF-Run4:RF  Run6:RF-Run4:RF  Run7:RF-Run4:RF  Run3:SHAM-Run4:RF  Run4:SHAM-Run4:RF  Run5:SHAM-Run4:RF  Run6:SHAM-Run4:RF  Run7:SHAM-Run4:RF  Run6:RF-Run5:RF  Run7:RF-Run5:RF  Run3:SHAM-Run5:RF  Run4:SHAM-Run5:RF  Run5:SHAM-Run5:RF  Run6:SHAM-Run5:RF  Run7:SHAM-Run5:RF  Run7:RF-Run6:RF  Run3:SHAM-Run6:RF  Run4:SHAM-Run6:RF  Run5:SHAM-Run6:RF  Run6:SHAM-Run6:RF  Run7:SHAM-Run6:RF  Run3:SHAM-Run7:RF  Run4:SHAM-Run7:RF  Run5:SHAM-Run7:RF  Run6:SHAM-Run7:RF  Run7:SHAM-Run7:RF  Run4:SHAM-Run3:SHAM  Run5:SHAM-Run3:SHAM  Run6:SHAM-Run3:SHAM  Run7:SHAM-Run3:SHAM  Run5:SHAM-Run4:SHAM  Run6:SHAM-Run4:SHAM  Run7:SHAM-Run4:SHAM  Run6:SHAM-Run5:SHAM  Run7:SHAM-Run5:SHAM  Run7:SHAM-Run6:SHAM | 0.043708158  0.041660602  -0.013938300  0.008367391  -0.089118790  -0.119763969  -0.054526028  -0.115693598  -0.101209471  -0.002047556  -0.057646458  -0.035340767  -0.132826948  -0.163472127  -0.098234186  -0.159401756  -0.144917629  -0.055598902  -0.033293211  -0.130779392  -0.161424571  -0.096186630  -0.157354200  -0.142870073  0.022305691  -0.075180490  -0.105825668  -0.040587728  -0.101755298  -0.087271171  -0.097486181  -0.128131359  -0.062893419  -0.124060989  -0.109576862  -0.030645178  0.034592762  -0.026574808  -0.012090681  0.065237940  0.004070370  0.018554497  -0.061167570  -0.046683443  0.014484127 | -0.02709974 0.114516055  -0.02914730 0.112468499  -0.08496012 0.057083524  -0.06277524 0.079510018  -0.15992669 -0.018310893  -0.19073787 -0.048790071  -0.12579089 0.016738830  -0.18652505 -0.044862151  -0.17204092 -0.030378024  -0.07306436 0.068969249  -0.12887656 0.013583646  -0.10669132 0.036009787  -0.20384375 -0.061810144  -0.23465444 -0.092289809  -0.16970662 -0.026761757  -0.23044204 -0.088361471  -0.21595791 -0.073877344  -0.12682901 0.015631202  -0.10464377 0.038057343  -0.20179620 -0.059762587  -0.23260689 -0.090242253  -0.16765906 -0.024714201  -0.22839449 -0.086313915  -0.21391036 -0.071829788  -0.04925717 0.093868551  -0.14641059 -0.003950386  -0.17722079 -0.034430545  -0.11227210 0.031096646  -0.17300881 -0.030501783  -0.15852469 -0.016017656  -0.16883674 -0.026135626  -0.19964665 -0.056616064  -0.13469748 0.008910643  -0.19543491 -0.052687064  -0.18095079 -0.038202937  -0.10182750 0.040537140  -0.03687967 0.106065192  -0.09761509 0.044465477  -0.08313097 0.058949604  -0.00639895 0.136874830  -0.06713537 0.075276114  -0.05265125 0.089760241  -0.13266333 0.010328191  -0.11817920 0.024812318  -0.05657963 0.085547885 | 0.6320  0.6949  0.9998  0.9999  0.0027  < 0.0001  0.3129  < 0.0001  0.0002  1.0000  0.2365  0.8638  < 0.0001  < 0.0001  0.0005  < 0.0001  < 0.0001  0.2848  0.9015  < 0.0001  < 0.0001  0.0008  < 0.0001  < 0.0001  0.9930  0.0288  0.0001  0.7406  0.0002  0.0042  0.0006  < 0.0001  0.1462  < 0.0001  < 0.0001  0.9384  0.8796  0.9749  0.9999  0.1107  1.0000  0.9982  0.1706  0.5515  0.9997 |
| Head | SHAM-RF | -0.04057167 | -0.05469012 -0.02645322 | < 0.0001 |
|  | Run4-Run3  Run5-Run3  Run6-Run3  Run7-Run3  Run5-Run4  Run6-Run4  Run7-Run4  Run6-Run5  Run7-Run5  Run7-Run6 | 0.009560395  0.007788876  0.015465065  0.011450543  -0.001771519  0.005904670  0.001890148  0.007676189  0.003661667  -0.004014521 | -0.02141735 0.04053814  -0.02320453 0.03878228  -0.01555983 0.04648996  -0.01960077 0.04250185  -0.03282687 0.02928383  -0.02518211 0.03699145  -0.02922299 0.03300329  -0.02342620 0.03877857  -0.02746706 0.03479040  -0.03517461 0.02714556 | 0.9174  0.9596  0.6533  0.8526  0.9998  0.9855  0.9998  0.9621  0.9977  0.9967 |
|  | Run4:RF-Run3:RF  Run5:RF-Run3:RF  Run6:RF-Run3:RF  Run7:RF-Run3:RF  Run3:SHAM-Run3:RF  Run4:SHAM-Run3:RF  Run5:SHAM-Run3:RF  Run6:SHAM-Run3:RF  Run7:SHAM-Run3:RF  Run5:RF-Run4:RF  Run6:RF-Run4:RF  Run7:RF-Run4:RF  Run3:SHAM-Run4:RF  Run4:SHAM-Run4:RF  Run5:SHAM-Run4:RF  Run6:SHAM-Run4:RF  Run7:SHAM-Run4:RF  Run6:RF-Run5:RF  Run7:RF-Run5:RF  Run3:SHAM-Run5:RF  Run4:SHAM-Run5:RF  Run5:SHAM-Run5:RF  Run6:SHAM-Run5:RF  Run7:SHAM-Run5:RF  Run7:RF-Run6:RF  Run3:SHAM-Run6:RF  Run4:SHAM-Run6:RF  Run5:SHAM-Run6:RF  Run6:SHAM-Run6:RF  Run7:SHAM-Run6:RF  Run3:SHAM-Run7:RF  Run4:SHAM-Run7:RF  Run5:SHAM-Run7:RF  Run6:SHAM-Run7:RF  Run7:SHAM-Run7:RF  Run4:SHAM-Run3:SHAM  Run5:SHAM-Run3:SHAM  Run6:SHAM-Run3:SHAM  Run7:SHAM-Run3:SHAM  Run5:SHAM-Run4:SHAM  Run6:SHAM-Run4:SHAM  Run7:SHAM-Run4:SHAM  Run6:SHAM-Run5:SHAM  Run7:SHAM-Run5:SHAM  Run7:SHAM-Run6:SHAM | -0.013381425  0.012885092  0.021446571  0.038201822  -0.034985318  -0.001706149  -0.032263972  -0.025243278  -0.050039473  0.026266517  0.034827995  0.051583246  -0.021603894  0.011675276  -0.018882547  -0.011861853  -0.036658048  0.008561478  0.025316729  -0.047870411  -0.014591241  -0.045149064  -0.038128370  -0.062924565  0.016755251  -0.056431889  -0.023152720  -0.053710543  -0.046689848  -0.071486044  -0.073187140  -0.039907971  -0.070465794  -0.063445099  -0.088241295  0.033279169  0.002721346  0.009742041  -0.015054155  -0.030557823  -0.023537128  -0.048333324  0.007020695  -0.017775501  -0.024796196 | -0.0637925752 0.0370297256  -0.0375764404 0.0633466247  -0.0291338943 0.0720270355  -0.0124647940 0.0888684372  -0.0857041127 0.0157334759  -0.0524950930 0.0490827947  -0.0830529161 0.0185249716  -0.0760146217 0.0255280665  -0.1008108173 0.0007318709  -0.0243271968 0.0768602306  -0.0158843407 0.0855403315  0.0007849833 0.1023815095  -0.0724542004 0.0292464131  -0.0392449995 0.0625955508  -0.0698028226 0.0320377277  -0.0627645736 0.0390408680  -0.0875607692 0.0142446724  -0.0422009408 0.0593238977  -0.0255315322 0.0761649911  -0.0987706648 0.0030298436  -0.0655613953 0.0363789127  -0.0961192184 0.0058210896  -0.0890809865 0.0128242471  -0.1138771822 -0.0119719485  -0.0342110406 0.0677215426  -0.1074500529 -0.0054137252  -0.0742406220 0.0279351826  -0.1047984452 -0.0026226405  -0.0977602537 0.0043805574  -0.1225564494 -0.0204156382  -0.1242907167 -0.0220835634  -0.0910811695 0.0112652280  -0.1216389926 -0.0192925951  -0.1146008304 -0.0122893680  -0.1393970260 -0.0370855636  -0.0179456921 0.0845040308  -0.0485035153 0.0539462076  -0.0414653706 0.0609494524  -0.0662615663 0.0361532567  -0.0818521421 0.0207364958  -0.0748140211 0.0277397642  -0.0996102167 0.0029435685  -0.0442561979 0.0582975873  -0.0690523936 0.0335013917  -0.0760556560 0.0264632647 | 0.9979  0.9984  0.9439  0.3342  0.4686  1.0000  0.5916  0.8611  0.0572  0.8269  0.4753  0.0430  0.9432  0.9993  0.9763  0.9992  0.4028  0.9999  0.8601  0.0860  0.9963  0.1353  0.3452  0.0037  0.9897  0.0168  0.9166  0.0301  0.1075  0.0004  0.0002  0.2860  0.0005  0.0034  < 0.0001  0.5590  1.0000  0.9998  0.9955  0.6792  0.9103  0.0844  0.9999  0.9851  0.8799 |

**Table S3.** The statistical outcomes for all the participants (n = 42) of a two-way mixed-effects model for the decomposed (CDA), conventional (TTP), and global measures of evoked EDA data, are displayed. The time period and effects of 5G were evaluated. Significant results (p-value < 0.05) are presented in green.

|  | **Time period between runs** | **5G exposure** | **Interaction** |
| --- | --- | --- | --- |
| **Continuous decomposition analysis (CDA)** | **F-value (DFn, DFd); p-value** | **F-value (DFn, DFd); p-value** | **F-value (DFn, DFd); p-value** |
| **CDA. Tonic** | F (4,794) = 34.79 ; p <0.0001 | F (1,797) = 32.56 ; p <0.0001 | F (4, 789) = 1.37 ; p = 0.2413 |
| **CDA. Phasic** | F (4, 784) = 1.53 ; p = 0.1895 | F (1, 1787) = 2.38 ; p = 0.1231 | F (4, 779) = 0.44 ; p = 0.7813 |
| **CDA.Latency** | F (4, 330) = 0.25 ; p = 0.9067 | F (1, 333) = 6.85 ; p = 0.0092 | F (4, 325) = 1.60 ; p = 0.1723 |
| **CDA.nCSR** | F (4, 794) = 1.33 ; p = 0.2566 | F (1, 797) = 2.64 ; p = 0.9999 | F (4, 789) = 0.74 ; p = 0.5616 |
| **CDA.AmpSum** | F (4, 794) = 1.18 ; p = 0.3179 | F (1, 797) = 0.02 ; p = 0.8742 | F (4, 789) = 0.48 ; p = 0.7465 |
| **Standard trough-to-peak (TTP) analysis** | **F (DFn, DFd); p-value** | **F (DFn, DFd); p-value** | **F (DFn, DFd); p-value** |
| **TTP.Latency** | F (4, 61) = 0.60 ; p = 0.6623 | F (1, 64) = 15.05 ; p = 0.0002 | F (4, 56) = 0.46 ; p = 0.7625 |
| **TTP.nSCR** | F (4, 794) = 0.78 ; p = 0.5326 | F (1, 797) = 0.96 ; p = 0.3274 | F (4, 789) = 0.32 ; p = 0.8617 |
| **TTP.AmpSum** | F (4, 739) = 1.20 ; p = 0.3093 | F (1, 742) = 0.56 ; p = 0.45339 | F (4, 734) = 0.86 ; p = 0.4825 |
| **Global Measures** | **F (DFn, DFd); p-value** | **F (DFn, DFd); p-value** | **F (DFn, DFd); p-value** |
| **Mean skin conductance (SC)** | F (4, 794) = 33.07 ; p <0.0001 | F (1, 797) = 31.60 ; p <0.0001 | F (4, 789) = 1.04 ; p = 0.3850 |
| **Maximum positive deflection** | F (4, 794) = 0.91 ; p = 0.4571 | F (1, 797) = 0.60 ; p = 0.4375 | F (4, 789) = 0.32 ; p = 0.8636 |

**Table S4.** The results of Tukey’s comparison test between the real “RF” and Sham exposure sessions for CDA.Tonic, CDA.Latency, TTP.Latency and global mean of SC, calculated from electrodermal activity recordings, are presented for the included volunteers (n = 42). Data from the first two stimulations were considered for this statistical test. Significant p-values (< 0.05) are highlighted in green (group comparisons between runs (time periods) within each session) and red (correspondent run comparison between the real “RF” and sham sessions). Abbreviations: CI, confidence interval.

| **Component** | **Tukey's multiple comparisons test** | **Mean Difference** | **95.00% CI of difference** | **Adjusted p-value** |
| --- | --- | --- | --- | --- |
| CDA.Latency | SHAM-RF | 0.2786091 | 0.08417807 0.4730402 | 0.0050 |
|  | Run4-Run3  Run5-Run3  Run6-Run3  Run7-Run3  Run5-Run4  Run6-Run4  Run7-Run4  Run6-Run5  Run7-Run5  Run7-Run6 | 0.043478808  0.049227258  -0.015304878  0.108989475  0.005748449  -0.058783687  0.065510667  -0.064532136  0.059762218  0.124294353 | -0.3981867 0.4851443  -0.3696018 0.4680563  -0.4232534 0.3926437  -0.3113448 0.5293238  -0.4459862 0.4574831  -0.5004492 0.3828818  -0.3876199 0.5186412  -0.4833612 0.3542969  -0.3711400 0.4906644  -0.2960399 0.5446286 | 0.9988  0.9976  0.9999  0.9539  0.9999  0.9962  0.9947  0.9933  0.9955  0.9272 |
|  | Run4:RF-Run3:RF  Run5:RF-Run3:RF  Run6:RF-Run3:RF  Run7:RF-Run3:RF  Run3:SHAM-Run3:RF  Run4:SHAM-Run3:RF  Run5:SHAM-Run3:RF  Run6:SHAM-Run3:RF  Run7:SHAM-Run3:RF  Run5:RF-Run4:RF  Run6:RF-Run4:RF  Run7:RF-Run4:RF  Run3:SHAM-Run4:RF  Run4:SHAM-Run4:RF  Run5:SHAM-Run4:RF  Run6:SHAM-Run4:RF  Run7:SHAM-Run4:RF  Run6:RF-Run5:RF  Run7:RF-Run5:RF  Run3:SHAM-Run5:RF  Run4:SHAM-Run5:RF  Run5:SHAM-Run5:RF  Run6:SHAM-Run5:RF  Run7:SHAM-Run5:RF  Run7:RF-Run6:RF  Run3:SHAM-Run6:RF  Run4:SHAM-Run6:RF  Run5:SHAM-Run6:RF  Run6:SHAM-Run6:RF  Run7:SHAM-Run6:RF  Run3:SHAM-Run7:RF  Run4:SHAM-Run7:RF  Run5:SHAM-Run7:RF  Run6:SHAM-Run7:RF  Run7:SHAM-Run7:RF  Run4:SHAM-Run3:SHAM  Run5:SHAM-Run3:SHAM  Run6:SHAM-Run3:SHAM  Run7:SHAM-Run3:SHAM  Run5:SHAM-Run4:SHAM  Run6:SHAM-Run4:SHAM  Run7:SHAM-Run4:SHAM  Run6:SHAM-Run5:SHAM  Run7:SHAM-Run5:SHAM  Run7:SHAM-Run6:SHAM | 0.031224564  0.140472030  -0.005573732  -0.167838781  0.207460942  0.264548917  0.155323382  0.186347919  0.593589147  0.109247466  -0.036798295  -0.199063345  0.176236378  0.233324353  0.124098818  0.155123355  0.562364583  -0.146045762  -0.308310811  0.066988912  0.124076887  0.014851351  0.045875889  0.453117117  -0.162265049  0.213034674  0.270122649  0.160897113  0.191921651  0.599162879  0.375299723  0.432387698  0.323162162  0.354186700  0.761427928  0.057087975  -0.052137561  -0.021113023  0.386128205  -0.109225536  -0.078200998  0.329040230  0.031024538  0.438265766  0.407241228 | -0.67694375 0.7393929  -0.53970973 0.8206538  -0.65602406 0.6448766  -0.84802054 0.5123430  -0.46328101 0.8782029  -0.46431777 0.9934156  -0.52485838 0.8355051  -0.48900621 0.8617020  -0.09165154 1.2788298  -0.62300994 0.8415049  -0.74152470 0.6679281  -0.93132075 0.5331941  -0.54726100 0.8997338  -0.54436474 1.0110134  -0.60815859 0.8563562  -0.57265194 0.8828987  -0.17459436 1.2993235  -0.82264326 0.5305517  -1.01353827 0.3969166  -0.62913846 0.7631163  -0.62821640 0.8763702  -0.69037611 0.7200788  -0.65469655 0.7464483  -0.25699087 1.1632251  -0.83886255 0.5143324  -0.45407230 0.8801416  -0.45540033 0.9956456  -0.51570038 0.8374946  -0.47982246 0.8636658  -0.08252015 1.2808459  -0.32082764 1.0714271  -0.31990558 1.1846810  -0.38206530 1.0283896  -0.34638574 1.0547591  0.05131995 1.4715359  -0.68668130 0.8008572  -0.74826493 0.6439898  -0.71252411 0.6702981  -0.31494304 1.0871995  -0.86151882 0.6430677  -0.82613225 0.6697303  -0.42783014 1.0859106  -0.66954790 0.7315970  -0.27184222 1.1483737  -0.29824394 1.1127264 | 1.0000  0.9997  1.0000  0.9987  0.9929  0.9782  0.9993  0.9970  0.1552  0.9999  1.0000  0.9973  0.9988  0.9943  0.9999  0.9996  0.3115  0.9995  0.9292  0.9999  0.9999  1.0000  1.0000  0.5773  0.9990  0.9911  0.9742  0.9990  0.9961  0.1409  0.7856  0.7158  0.9071  0.8429  0.0245  0.9999  1.0000  1.0000  0.7637  0.9999  0.9999  0.9315  1.0000  0.6243  0.7106 |
| CDA.Tonic | SHAM-RF | 0.6129913 | 0.3585133 0.8674693 | < 0.0001 |
|  | Run4-Run3  Run5-Run3  Run6-Run3  Run7-Run3  Run5-Run4  Run6-Run4  Run7-Run4  Run6-Run5  Run7-Run5  Run7-Run6 | -1.2877911  -1.1686375  0.1842702  -0.7137982  0.1191536  1.4720613  0.5739929  1.3529077  0.4548393  -0.8980685 | -1.84819675 -0.7273854  -1.72904318 -0.6082318  -0.37613544 0.7446759  -1.27420389 -0.1533925  -0.44125211 0.6795592  0.91165563 2.0324670  0.01358718 1.1343985  0.79250206 1.9133134  -0.10556639 1.0152450  -1.45847413 -0.3376628 | < 0.0001  < 0.0001  0.8973  0.0047  0.9778  < 0.0001  0.0416  < 0.0001  0.1736  0.0001 |
|  | Run4:RF-Run3:RF  Run5:RF-Run3:RF  Run6:RF-Run3:RF  Run7:RF-Run3:RF  Run3:SHAM-Run3:RF  Run4:SHAM-Run3:RF  Run5:SHAM-Run3:RF  Run6:SHAM-Run3:RF  Run7:SHAM-Run3:RF  Run5:RF-Run4:RF  Run6:RF-Run4:RF  Run7:RF-Run4:RF  Run3:SHAM-Run4:RF  Run4:SHAM-Run4:RF  Run5:SHAM-Run4:RF  Run6:SHAM-Run4:RF  Run7:SHAM-Run4:RF  Run6:RF-Run5:RF  Run7:RF-Run5:RF  Run3:SHAM-Run5:RF  Run4:SHAM-Run5:RF  Run5:SHAM-Run5:RF  Run6:SHAM-Run5:RF  Run7:SHAM-Run5:RF  Run7:RF-Run6:RF  Run3:SHAM-Run6:RF  Run4:SHAM-Run6:RF  Run5:SHAM-Run6:RF  Run6:SHAM-Run6:RF  Run7:SHAM-Run6:RF  Run3:SHAM-Run7:RF  Run4:SHAM-Run7:RF  Run5:SHAM-Run7:RF  Run6:SHAM-Run7:RF  Run7:SHAM-Run7:RF  Run4:SHAM-Run3:SHAM  Run5:SHAM-Run3:SHAM  Run6:SHAM-Run3:SHAM  Run7:SHAM-Run3:SHAM  Run5:SHAM-Run4:SHAM  Run6:SHAM-Run4:SHAM  Run7:SHAM-Run4:SHAM  Run6:SHAM-Run5:SHAM  Run7:SHAM-Run5:SHAM  Run7:SHAM-Run6:SHAM | -1.40442738  -1.28604286  0.05357976  -1.06941190  0.32485298  -0.84630179  -0.72637917  0.63981369  -0.03333155  0.11838452  1.45800714  0.33501548  1.72928036  0.55812560  0.67804821  2.04424107  1.37109583  1.33962262  0.21663095  1.61089583  0.43974107  0.55966369  1.92585655  1.25271131  -1.12299167  0.27127321  -0.89988155  -0.77995893  0.58623393  -0.08691131  1.39426488  0.22311012  0.34303274  1.70922560  1.03608036  -1.17115476  -1.05123214  0.31496071  -0.35818452  0.11992262  1.48611548  0.81297024  1.36619286  0.69304762  -0.67314524 | -2.3241046 -0.48475016  -2.2057201 -0.36636564  -0.8660975 0.97325698  -1.9890891 -0.14973469  -0.5948242 1.24453020  -1.7659790 0.07337543  -1.6460564 0.19329805  -0.2798635 1.55949091  -0.9530088 0.88634567  -0.8012927 1.03806174  0.5383299 2.37768436  -0.5846617 1.25469270  0.8096031 2.64895758  -0.3615516 1.47780281  -0.2416290 1.59772543  1.1245639 2.96391829  0.4514186 2.29077305  0.4199454 2.25929984  -0.7030463 1.13630817  0.6912186 2.53057305  -0.4799361 1.35941829  -0.3600135 1.47934091  1.0061793 2.84553377  0.3330341 2.17238853  -2.0426689 -0.20331445  -0.6484040 1.19095043  -1.8195588 0.01979567  -1.6996361 0.13971829  -0.3334433 1.50591115  -1.0065885 0.83276591  0.4745877 2.31394210  -0.6965671 1.14278734  -0.5766445 1.26270996  0.7895484 2.62890281  0.1164031 1.95575758  -2.0908320 -0.25147754  -1.9709094 -0.13155492  -0.6047165 1.23463793  -1.2778617 0.56149270  -0.7997546 1.03959984  0.5664383 2.40579270  -0.1067070 1.73264746  0.4465156 2.28587008  -0.2266296 1.61272484  -1.5928225 0.24653198 | < 0.0001  0.0004  1.0000  0.0090  0.9826  0.1018  0.2668  0.4527  1.0000  0.9999  < 0.0001  0.9785  < 0.0001  0.6517  0.3649  < 0.0001  0.0001  0.0001  0.9991  < 0.0001  0.8854  0.6481  < 0.0001  0.0007  0.0045  0.9952  0.0611  0.1791  0.5835  0.9999  < 0.0001  0.9989  0.9748  < 0.0001  0.0136  0.0023  0.0113  0.9859  0.9664  0.9999  < 0.0001  0.1365  0.0001  0.3327  0.3757 |
| TTP.Latency | SHAM-RF | 0.5436307 | 0.04924437 1.038017 | 0.0315 |
|  | Run4-Run3  Run5-Run3  Run6-Run3  Run7-Run3  Run5-Run4  Run6-Run4  Run7-Run4  Run6-Run5  Run7-Run5  Run7-Run6 | -0.47121429  -0.13831366  0.16536786  0.03014286  0.33290062  0.63658214  0.50135714  0.30368152  0.16845652  -0.13522500 | -1.6115391 0.6691106  -1.1358323 0.8592049  -0.8672402 1.1979759  -1.1362004 1.1964861  -0.7874170 1.4532182  -0.5150895 1.7882538  -0.7716019 1.7743162  -0.7067888 1.3141518  -0.9783335 1.3152465  -1.3126643 1.0422143 | 0.7777  0.9951  0.9915  0.9999  0.9208  0.5384  0.8065  0.9177  0.9939  0.9976 |
|  | Run4:RF-Run3:RF  Run5:RF-Run3:RF  Run6:RF-Run3:RF  Run7:RF-Run3:RF  Run3:SHAM-Run3:RF  Run4:SHAM-Run3:RF  Run5:SHAM-Run3:RF  Run6:SHAM-Run3:RF  Run7:SHAM-Run3:RF  Run5:RF-Run4:RF  Run6:RF-Run4:RF  Run7:RF-Run4:RF  Run3:SHAM-Run4:RF  Run4:SHAM-Run4:RF  Run5:SHAM-Run4:RF  Run6:SHAM-Run4:RF  Run7:SHAM-Run4:RF  Run6:RF-Run5:RF  Run7:RF-Run5:RF  Run3:SHAM-Run5:RF  Run4:SHAM-Run5:RF  Run5:SHAM-Run5:RF  Run6:SHAM-Run5:RF  Run7:SHAM-Run5:RF  Run7:RF-Run6:RF  Run3:SHAM-Run6:RF  Run4:SHAM-Run6:RF  Run5:SHAM-Run6:RF  Run6:SHAM-Run6:RF  Run7:SHAM-Run6:RF  Run3:SHAM-Run7:RF  Run4:SHAM-Run7:RF  Run5:SHAM-Run7:RF  Run6:SHAM-Run7:RF  Run7:SHAM-Run7:RF  Run4:SHAM-Run3:SHAM  Run5:SHAM-Run3:SHAM  Run6:SHAM-Run3:SHAM  Run7:SHAM-Run3:SHAM  Run5:SHAM-Run4:SHAM  Run6:SHAM-Run4:SHAM  Run7:SHAM-Run4:SHAM  Run6:SHAM-Run5:SHAM  Run7:SHAM-Run5:SHAM  Run7:SHAM-Run6:SHAM | -0.29866667  -0.04855303  0.55243333  -0.11591667  0.87952778  0.17825000  0.50187500  0.53218333  0.85536905  0.25011364  0.85110000  0.18275000  1.17819444  0.47691667  0.80054167  0.83085000  1.15403571  0.60098636  -0.06736364  0.92808081  0.22680303  0.55042803  0.58073636  0.90392208  -0.66835000  0.32709444  -0.37418333  -0.05055833  -0.02025000  0.30293571  0.99544444  0.29416667  0.61779167  0.64810000  0.97128571  -0.70127778  -0.37765278  -0.34734444  -0.02415873  0.32362500  0.35393333  0.67711905  0.03030833  0.35349405  0.32318571 | -2.0576724 1.460339  -1.6572151 1.560109  -1.0976603 2.202527  -2.0428109 1.810978  -0.8198332 2.578889  -1.7486443 2.105144  -1.0714276 2.075178  -1.1179103 2.182277  -0.9774730 2.688211  -1.5405885 2.040816  -0.9769124 2.679112  -1.8985337 2.264034  -0.6944102 3.050799  -1.6043670 2.558200  -0.9584641 2.559547  -0.9971624 2.658862  -0.8404893 3.148561  -1.0828550 2.284828  -2.0232354 1.888508  -0.8040683 2.660230  -1.7290687 2.182675  -1.0582341 2.159090  -1.1031050 2.264578  -0.9593607 2.767205  -2.6584378 1.321738  -1.4435992 2.097788  -2.3642712 1.615904  -1.7006520 1.599535  -1.7437166 1.703217  -1.5962320 2.202103  -1.0356804 3.026569  -1.9308192 2.519153  -1.3091026 2.544686  -1.3419878 2.638188  -1.1727643 3.115336  -2.7324027 1.329847  -2.0770138 1.321708  -2.1180381 1.423349  -1.9662856 1.917968  -1.6032693 2.250519  -1.6361545 2.344021  -1.4669310 2.821169  -1.6197853 1.680402  -1.4793480 2.186336  -1.5759820 2.222353 | 0.9999  1.0000  0.9844  1.0000  0.8011  0.9999  0.9889  0.9880  0.8808  0.9999  0.8823  0.9999  0.5692  0.9990  0.8958  0.8965  0.6805  0.9760  1.0000  0.7674  0.9999  0.9819  0.9809  0.8545  0.9841  0.9998  0.9998  1.0000  1.0000  0.9999  0.8469  0.9999  0.9885  0.9871  0.8984  0.9808  0.9992  0.9997  1.0000  0.9999  0.9998  0.9896  1.0000  0.9997  0.9999 |
| Global.Mean | SHAM-RF | 0.6496283 | 0.3727279 0.9265288 | < 0.0001 |
|  | Run4-Run3  Run5-Run3  Run6-Run3  Run7-Run3  Run5-Run4  Run6-Run4  Run7-Run4  Run6-Run5  Run7-Run5  Run7-Run6 | -1.3601601  -1.2204929  0.1938333  -0.7629786  0.1396673  1.5539935  0.5971815  1.4143262  0.4575143  -0.9568119 | -1.96994385 -0.7503764  -1.83027659 -0.6107091  -0.41595040 0.8036171  -1.37276230 -0.1531948  -0.47011647 0.7494510  0.94420972 2.1637772  -0.01260218 1.2069653  0.80454246 2.0241099  -0.15226945 1.0672980  -1.56659564 -0.3470282 | < 0.0001  < 0.0001  0.9081  0.0058  0.9708  < 0.0001  0.0582  < 0.0001  0.2426  0.0001 |
|  | Run4:RF-Run3:RF  Run5:RF-Run3:RF  Run6:RF-Run3:RF  Run7:RF-Run3:RF  Run3:SHAM-Run3:RF  Run4:SHAM-Run3:RF  Run5:SHAM-Run3:RF  Run6:SHAM-Run3:RF  Run7:SHAM-Run3:RF  Run5:RF-Run4:RF  Run6:RF-Run4:RF  Run7:RF-Run4:RF  Run3:SHAM-Run4:RF  Run4:SHAM-Run4:RF  Run5:SHAM-Run4:RF  Run6:SHAM-Run4:RF  Run7:SHAM-Run4:RF  Run6:RF-Run5:RF  Run7:RF-Run5:RF  Run3:SHAM-Run5:RF  Run4:SHAM-Run5:RF  Run5:SHAM-Run5:RF  Run6:SHAM-Run5:RF  Run7:SHAM-Run5:RF  Run7:RF-Run6:RF  Run3:SHAM-Run6:RF  Run4:SHAM-Run6:RF  Run5:SHAM-Run6:RF  Run6:SHAM-Run6:RF  Run7:SHAM-Run6:RF  Run3:SHAM-Run7:RF  Run4:SHAM-Run7:RF  Run5:SHAM-Run7:RF  Run6:SHAM-Run7:RF  Run7:SHAM-Run7:RF  Run4:SHAM-Run3:SHAM  Run5:SHAM-Run3:SHAM  Run6:SHAM-Run3:SHAM  Run7:SHAM-Run3:SHAM  Run5:SHAM-Run4:SHAM  Run6:SHAM-Run4:SHAM  Run7:SHAM-Run4:SHAM  Run6:SHAM-Run5:SHAM  Run7:SHAM-Run5:SHAM  Run7:SHAM-Run6:SHAM | -1.43796310  -1.32903452  0.09294405  -1.08990476  0.40396429  -0.87839286  -0.70798690  0.69868690  -0.03208810  0.10892857  1.53090714  0.34805833  1.84192738  0.55957024  0.72997619  2.13665000  1.40587500  1.42197857  0.23912976  1.73299881  0.45064167  0.62104762  2.02772143  1.29694643  -1.18284881  0.31102024  -0.97133690  -0.80093095  0.60574286  -0.12503214  1.49386905  0.21151190  0.38191786  1.78859167  1.05781667  -1.28235714  -1.11195119  0.29472262  -0.43605238  0.17040595  1.57707976  0.84630476  1.40667381  0.67589881  -0.73077500 | -2.43867424 -0.43725195  -2.32974567 -0.32832338  -0.90776709 1.09365519  -2.09061590 -0.08919362  -0.59674686 1.40467543  -1.87910400 0.12231828  -1.70869805 0.29272424  -0.30202424 1.69939805  -1.03279924 0.96862305  -0.89178257 1.10963971  0.53019600 2.53161828  -0.65265281 1.34876948  0.84121624 2.84263852  -0.44114090 1.56028138  -0.27073495 1.73068733  1.13593886 3.13736114  0.40516386 2.40658614  0.42126743 2.42268971  -0.76158138 1.23984090  0.73228767 2.73370995  -0.55006948 1.45135281  -0.37966352 1.62175876  1.02701029 3.02843257  0.29623529 2.29765757  -2.18355995 -0.18213767  -0.68969090 1.31173138  -1.97204805 0.02937424  -1.80164209 0.19978019  -0.39496828 1.60645400  -1.12574328 0.87567900  0.49315791 2.49458019  -0.78919924 1.21222305  -0.61879328 1.38262900  0.78788052 2.78930281  0.05710552 2.05852781  -2.28306828 -0.28164600  -2.11266233 -0.11124005  -0.70598852 1.29543376  -1.43676352 0.56465876  -0.83030519 1.17111709  0.57636862 2.57779090  -0.15440638 1.84701590  0.40596267 2.40738495  -0.32481233 1.67660995  -1.73148614 0.26993614 | 0.0002  0.0011  0.9999  0.0204  0.9578  0.1433  0.4270  0.4472  1.0000  0.9999  < 0.0001  0.9843  < 0.0001  0.7516  0.3808  < 0.0001  0.0004  0.0003  0.9990  < 0.0001  0.9181  0.6213  < 0.0001  0.0017  0.0072  0.9930  0.0656  0.2492  0.6552  0.9999  0.0001  0.9996  0.9706  < 0.0001  0.0285  0.0021  0.0161  0.9953  0.9325  0.9999  < 0.0001  0.1822  0.0003  0.4976  0.3791 |
